# A compact ultra-wideband bandstop filter using mu-negative and ENZ metamaterial resonators for multiple advanced wireless communication systems

**DOI:** 10.1371/journal.pone.0335521

**Published:** 2025-11-06

**Authors:** Mouhssine Elbathaoui, Nawfal Jebbor, Sudipta Das, Wael Ali, Sahaya Anselin Nisha Arockiam, Om Prakash Kumar, El-Mahjoub Boufounas

**Affiliations:** 1 ER2TI Laboratory, Engineering Science Department, Faculty of Sciences and Techniques, Modeling, Control of Optoelectronic Systems and Components MCSCO, Moulay Ismail University of Meknes, Errachidia, Morocco; 2 Electronics Instrumentation and Intelligent Systems Team, ER2TI Laboratory, Engineering Science Department, Faculty of Sciences and Techniques, Moulay Ismail University of Meknes, Errachidia, Morocco; 3 Department of Electronics and Communication Engineering, IMPS College of Engineering and Technology, Malda, West Bengal, India; 4 Department of Electronics & Communications Engineering, College of Engineering and Technology, Arab Academy for Science, Technology and Maritime Transport (AASTMT), Alexandria, Egypt; 5 Department of Electronics and Communication Engineering, Sathyabama Institute of Science and Technology, Chennai, Tamil Nadu, India; 6 Department of Electronics and Communication Engineering, Manipal Institute of Technology, Manipal Academy of Higher Education, Manipal, 576104, Karnataka, India; Parul University Parul Institute of Technology, INDIA

## Abstract

A compact ultra-wideband bandstop filter (UWB-BSF) based on a microstrip line and innovative complementary electrical LC (CELC)-loaded metamaterial (MTM) resonators is proposed. These MTM resonators exhibit negative effective permeability and epsilon-near-zero (ENZ) effective permittivity. The design employs a Rogers RO3006™ substrate and achieves an ultra-wide 3-dB stopband extending from 5.4 GHz to 21.6 GHz, equivalent to a fractional bandwidth of 120%, with a high rejection level. The shape factor (SF = 0.87), close to 1, and seven transmission zeros indicate high selectivity in the transition bands. The group delay remains flat in the lower and upper passbands, with GD ≤ 0.74 ns and GD ≤ 0.50 ns, respectively. The suggested UWB-BSF, with an overall size of 0.55λg × 2.22λg × 0.07λg, has been validated through simulations and measurements. The results demonstrate significant selectivity in the transition bands, making the filter particularly suitable for modern technologies such as 5G (Sub-6 GHz) and 5G NR (New Radio), especially within the n77 (3.3–4.2 GHz), n78 (3.3–3.8 GHz), and n79 (4.4–5.0 GHz) frequency bands, as well as Wi-Fi 5 (802.11ac), Wi-Fi 6 (802.11ax), 4G LTE, Ku-band satellite communications and K-band radar systems, by enhancing precision through effective mitigation of undesired signals. This research supports SDG 9, SDG 11, and SDG 12 by fostering innovation in wireless communication infrastructure, enabling sustainable smart city applications, and promoting efficient, compact design practices.

## 1. Introduction

Microwave band-stop filters (BSFs) have played a crucial role in the recent evolution of wireless communication systems [1]. Their ability to selectively attenuate unwanted frequencies while allowing the passage of desired frequencies, particularly within the RF/microwave range spanning 300 MHz to 300 GHz, has been extensively leveraged in modern wireless systems. The radio spectrum is a valuable and limited resource for implementing wireless devices/systems. The efficient utilization of the radio spectrum without causing interference among users leads to optimized spectrum usage in modern wireless communication systems. The bandstop filters used in communication systems are characterized by compact size, low footprint, ease of integration, efficient attenuation of stopbands, reliable suppression of interference and harmonics, and an extended stopband. These features meet the growing demands of emerging technologies such as 5G systems [2,3]. These filters are also well-suited for applications targeting the 5G NR (New Radio), especially within the n77/n78/n79 frequency bands and WLAN (5.8 GHz) bands, as well as the LTE 46 band [4]. Furthermore, their use in satellite systems operating in the K-band (18–26 GHz) ensures wide bandwidths and robust attenuation [5].

Different techniques have been adopted to design bandstop filters by implementing various methodologies. Among these, methods based on the use of open stub resonators and coupled lines have been widely explored [6–9], as well as solutions relying on waveguide elements [10] and substrate-integrated waveguides (SIWs) [11]. An ultra-wideband (UWB) tunable bandstop filter has been developed by integrating a varactor diode, open stubs, and stepped impedance resonators (SIRs). This filter features a UWB response covering the 2.44 GHz to 9.65 GHz band, including five transmission zeros (TZs) and an adjustable range of 1 GHz (8.65 GHz to 9.65 GHz) [12]. In addition, a UWB-BSF design utilizing a single-section coupled line, combined with a modified open stub (OCS) and parallel-connected unit cells, has been proposed. This structure improves selectivity and ensures device symmetry, reaching a relative stopband bandwidth of 92.62% (6.5 GHz to 23 GHz) [13]. Another method, based on frequency-selective surfaces (FSS), has been explored in [14]. This method prints two analogous metal layers on either side of an RO4003 substrate. Two designs are produced: the first provides a wide stopband of 9.8 GHz in the X and Ku bands, while the second achieves a stopband of 33.5 GHz in the millimeter band. A dual-band bandstop filter design utilizing planar linked lines in conjunction with grounded stepped impedance sections and a resistor R has been delineated [15]. The first and second-stop bands feature a −10 dB rejection bandwidth of 30.42% (0.53 GHz to 0.71 GHz) and 13.87% (1.26 GHz to 1.44 GHz), respectively, with a peak rejection level of −40.45 dB at 1.33 GHz. An innovative plasmonic approach has also been explored to design a bandstop filter based on surface plasmon-polaritons (SPP). This structure, composed of a metal-insulator-metal waveguide (MIM WG) and symmetrical semi-circular resonators containing silver nanorods (AgND), enables a tunable wide bandgap extending up to 2104 nm. In addition, this design can be used as a nanosensor, with a sensitivity of 7980 nm/RIU [16]. This innovative plasmonic approach is mainly suited to optical and nanometric applications, but remains limited for use in traditional microwave systems. A miniature bandstop filter has been realized by implementing complementary split-ring resonator (CSRR) metamaterials, connected in series with capacitive gaps [17]. These CSRRs enable CMOS compatibility and offer band rejection of up to 40 dB in stop bands. Additionally, a complementary Z-shaped electrical metamaterial resonator (CELC) design has a wide stopband with sharp transitions [18]. The filter exhibits an insertion loss bandwidth of 2.3 GHz (from 6.7 GHz to 9 GHz) at a 3 dB level, with a rejection exceeding 20 dB between 7.5 and 8.2 GHz.

Some designs of these reported BSFs have disadvantages that can reduce their performance and applicability. In particular, an insufficiently wide rejection band is often observed. At the same time, designs based on open stub resonators and coupled lines do not always guarantee clean transitions or high rejection over the entire stopband. Tunable UWB filters, despite their adjustable range, are characterized by reduced selectivity, mainly due to the complexity of stepped impedance resonators and the integration of varactor diodes. In addition, methods incorporating substrate-integrated waveguides (SIWs) suffer from significant structural complexity, sometimes requiring bulky structures that complicate their integration into compact systems. Meanwhile, filters using frequency-selective surfaces (FSS) require complex panels, increasing manufacturing costs and assembly time. However, the proposed solutions often lack efficiency in the high-frequency bands, where less sharp transition bands and reduced selectivity are frequently observed.

In this work, an ultra-wideband bandstop filter (UWB-BSF) has been designed using a complementary electrical LC (CELC)-loaded metamaterial (MTM) resonators, which exhibit negative effective permeability and epsilon-near-zero (ENZ) effective permittivity. This resonator has been modeled using localized LC elements and analyzed using the High-Frequency Structure Simulator (HFSS) tool. The proposed filter offers several advantages, featuring an extensive stopband, narrow transition bands, high rejection, and significant compactness. The measurements of the designed UWB-BSF offer a −3 dB bandwidth extending from 5.4 GHz to 21.6 GHz, with a maximum attenuation of −62.96 dB. The relative stopband bandwidth (RSB) at 3 dB is 120%, and the filter features seven transmission zeros. Its compact size is 0.55λg × 2.22λg × 0.07λg, with a shape factor of 0.87, indicating high selectivity. The filter offers outstanding performance in efficiently isolating desired frequencies while blocking unwanted frequencies and harmonics, making it particularly suitable for Sub-6GHz 5G NR (New Radio) applications, including n77 (3.3–4.2 GHz), n78 (3.3–3.8 GHz), and n79 (4.4–5.0 GHz) frequency bands and Bluetooth, Wi-Fi 5 (802.11ac), Wi-Fi 6 (802.11ax), 4G LTE band 46, Ku-band satellite communications, and K-band radar systems, where advanced filtering capabilities are required. The innovative MTM CELC resonator used in this design has considerable potential for developing various devices that require compact dimensions and efficient attenuation.

**The key contributions are listed as follows:**

i Introduction of a compact ultra-wideband bandstop filter (UWB-BSF) utilizing CELC-loaded metamaterial resonators with negative effective permeability and epsilon-near-zero (ENZ) effective permittivity.
ii A comprehensive analysis of the UWB-BSF model using numerical simulation via the HFSS tool, equivalent circuit modeling using the ADS tool, and experimental validation by testing the prototype with a VNA instrument.
iii The designed UWB-BSF achieves a measured 3-dB stopband ranging from 5.4 GHz to 21.6 GHz, demonstrating an ultrawide bandstop with an impressive relative stopband bandwidth of 120% and a high rejection level.
  iv The UWB-BSF exhibits exceptional selectivity, with a shape factor (SF) of 0.87 and seven transmission zeros, enhancing performance in transition bands.
    v Extremely compact design with dimensions of 0.55λg × 2.22λg × 0.07λg and flat group delay in the lower and upper passbands (≤ 0.74 ns and ≤ 0.50 ns, respectively).
  vi It is suitable for several modern wireless technologies, including 5G (Sub-6 GHz), 5G NR (n77, n78, n79 bands), Bluetooth, Wi-Fi 5/6, 4G LTE 46, Ku-band satellite communications, and K-band radar systems, offering effective rejection of unwanted signals.

## 2. Analysis of the metamaterial unit cell proposed with CELC loading

### 2.1. Structure and corresponding circuit model

**Fig 1:**
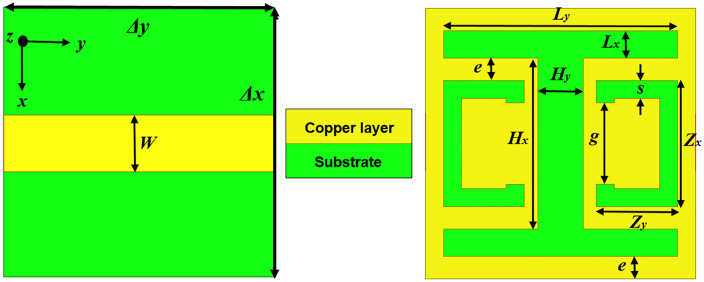
Design layout of the MTM unit cell with CELC resonator.

Fig 1 illustrates the structure of the MTM (metamaterial) unit cell. This MTM unit cell is made up of a copper microstrip line of width (*W*) on the top layer and a CELC (complementary electrical LC) resonator etched into the bottom ground plane layer. It is patterned into an I-shape and two opposing C-shapes. A Rogers RO3006™ substrate is used, with a relative permittivity of 6.15, 0.762 mm thickness, and a dielectric dissipation factor of 0.0025. The horizontal arms of the incorporated slot are defined by the dimensions *L*_*x*_ and *L*_*y*_, while *H*_*x*_ and *H*_*y*_ characterize the central vertical arms. The geometric parameters *Zx*, *Zy*, *s*, and *g* are specifically selected to optimize the bandstop performance of the CELC, describing the two complementary split rings.

**Fig 2:**
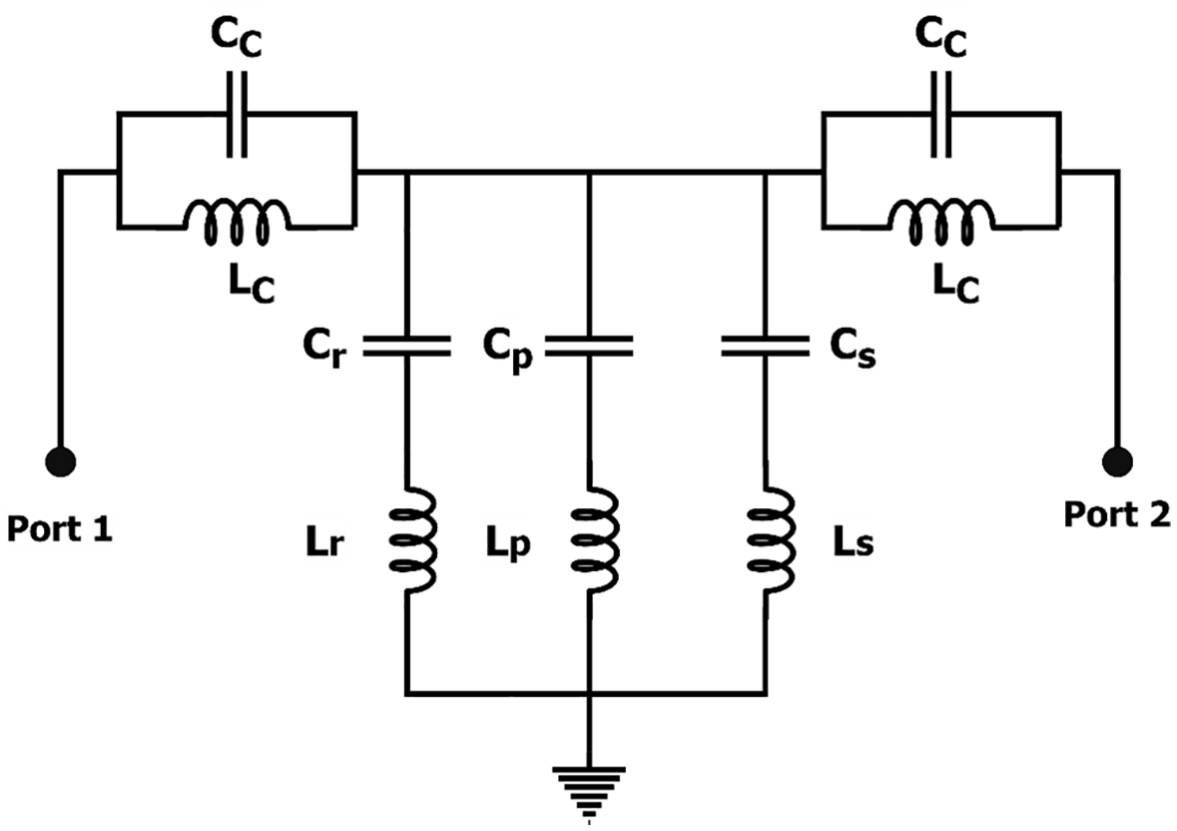
ECM of the MTM unit cell $\begin{array}{cc} & {(𝐂}_{C}=\text{240}.\text{798 fF},\;C_{r}=\text{45}.\text{0704 fF},C_{P}=\text{981}.\text{285 fF},{C_{S}=\text{88}.\text{8388 fF},\;L}_{C}=\text{759}.\text{985 pH},\;L_{r}=\text{777}.\text{171 pH}, \end{array}$$L_{P}=\text{349}.\text{563 pH},\;L_{S}=\text{955}.\text{317 pH})$.

The electrical equivalent circuit model with lumped elements is depicted in Fig 2. This model uses *L*_*p*_ to represent the microstrip line’s inductance, and *C*ₚ** to represent its capacitance and coupling with the ground plane’s CELC resonator. The complementary split rings in the ground plane are modeled by *L*_*C*_ inductances and *C*_*C*_ capacitances. The components *L*ₛ** and *C*ₛ** correspond respectively to the inductance and capacitance generated by the vertical arm, while *L*ᵣ** and *C*ᵣ** represent the inductive and capacitive elements produced by the horizontal arms. It should be noted that *L*ₚ**, *L*ᵣ*,* and *L*ₛ** result from the inductive effect of current flowing along the CELC slot edges.

**Fig 3:**
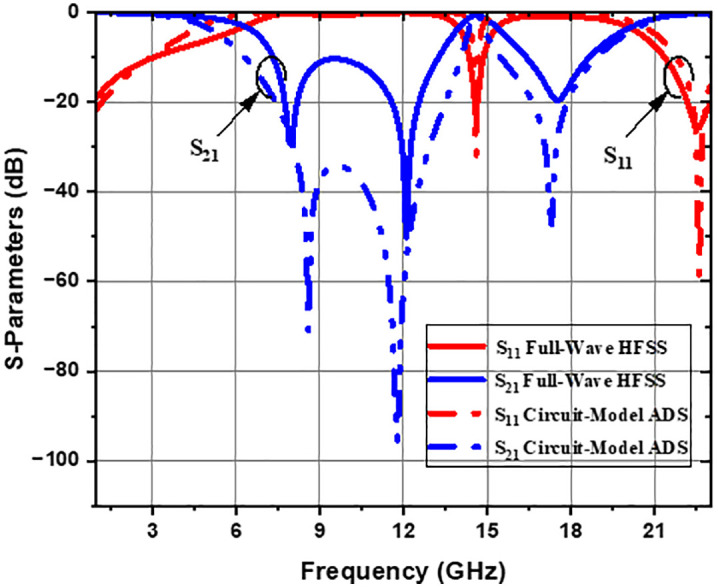
Simulation results of the S-parameters of the CELC-based MTM cell.

The suggested lumped-element circuit model is validated using planar electromagnetic (EM) and electrical simulations to evaluate the CELC subwavelength resonator. Circuit parameters are extracted in the Advanced Design System (ADS) simulator. Fig 3 shows the frequency response of the S-parameters of the CELC integrated metamaterial cell (MC), simulated with the High-Frequency Structure Simulator (HFSS) software, as well as that obtained for the equivalent circuit model using the ADS tool. The values of the capacitive and inductive components of the equivalent circuit model (ECM) are obtained with ADS after fixing the initial schematic, an optimization is performed to ensure agreement between the S_11_ and S_21_ parameters of the ECM and those simulated in HFSS, using the “Goal” option with limit lines defining the frequency intervals [$f_{min}$, $f_{max}$], the resonance frequencies, and the corresponding values of the S-parameters in decibels. The capacitances are constrained between $C_{min}=\text{1}\text{0}\text{ fF}$ and $C_{max}=\text{1}\text{0}\text{0}\text{0}\text{ fF}$, while the inductances are limited to $L_{min}=\text{1}\text{0}\text{ pH}$ and $L_{min}=\text{1}\text{0}\text{0}\text{0}\text{ pH}$, to account for the very small values required at the high frequencies under study. The optimization is carried out through the “Simulation Variables Setup (Optimization)” option, in Random mode with 1000 iterations, enabling ADS to provide the optimal values of the ECM components automatically, ensuring accurate reproduction of the observed electromagnetic results. The S-parameters derived from the EM and electrical simulations show good agreement. The slight discrepancy observed is mainly attributable to the wide frequency band considered, as circuit models lose accuracy in describing EM phenomena when the band exceeds the appropriate frequency range. The S-parameters reveal the presence of three transmission zeros around the frequencies of 8 GHz, 12 GHz, and 17.6 GHz, as well as three bandwidths observed between 0–6 GHz, 14 to 15.4 GHz, and 20–23 GHz.

### 2.2. Effective electromagnetic parameters of the MTM unit cell loaded with CELC

Metamaterials such as mu-negative (MNG) resonators [19,20] and epsilon-negative (ENG) resonators [21] have shown increasing interest in their unique properties. In this study, the proposed CELC resonator exhibits a μ-negative magnetic response, thus qualifying it as a mu (μ)-negative metamaterial. This feature makes the resonator particularly attractive for applications requiring a high capacity to attenuate unwanted frequency bands [22]. The evanescent modes generated by CELC resonator metamaterials can be described by equation (1) [18].


$$k^{\text{2}}=-\left(\beta^{\text{2}}\;+\omega^{\text{2}}\varepsilon \mu \right)$$ (1)

The wave vector, denoted k, represents wave propagation, while β characterizes the phase coefficient. When the permeability is negative (μ<0) and permittivity is positive (ε>0), it reveals a wave vector with an imaginary component. The effective electromagnetic attributes of the proposed CELC resonator-integrated MTM cell are derived from equations (2), (3), (4), and (5) [23] using the S parameters.


$$Z=\sqrt{\frac{{\left(\text{1}+S_{\text{1}\text{1}}\right)}^{\text{2}}-S_{\text{2}\text{1}}^{\text{2}}}{{\left(\text{1}-S_{\text{1}\text{1}}\right)}^{\text{2}}-S_{\text{2}\text{1}}^{\text{2}}}}$$ (2)

$$n=\;\frac{\text{1}}{kd}\text{ arccos}\left(\frac{\text{1}-S_{\text{1}\text{1}}^{\text{2}}+S_{\text{2}\text{1}}^{\text{2}}}{\text{2}S_{\text{2}\text{1}}}\right)$$ (3)

μ=n×Z (4)

$$\varepsilon =\frac{n}{Z}$$ (5)

**Fig 4:**
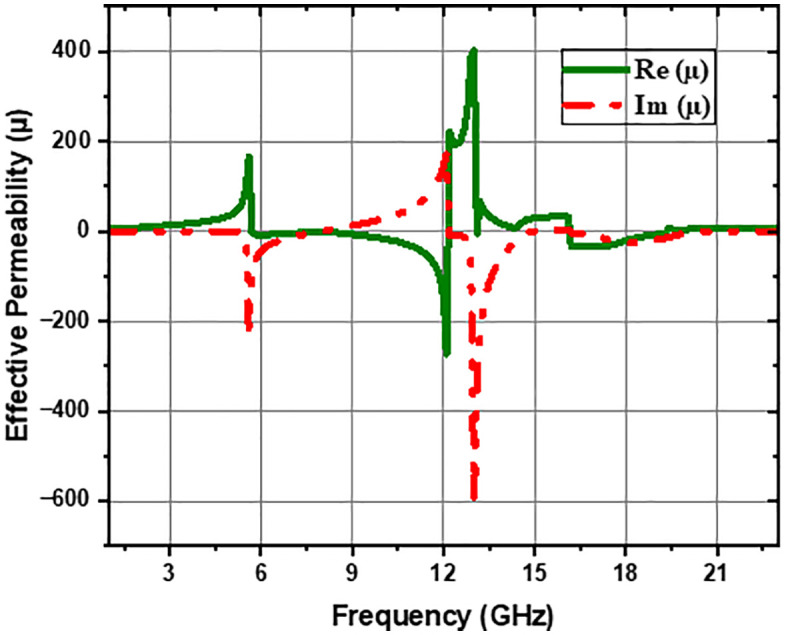
Effective permeability of the MTM unit cell loaded with CELC.

**Fig 5:**
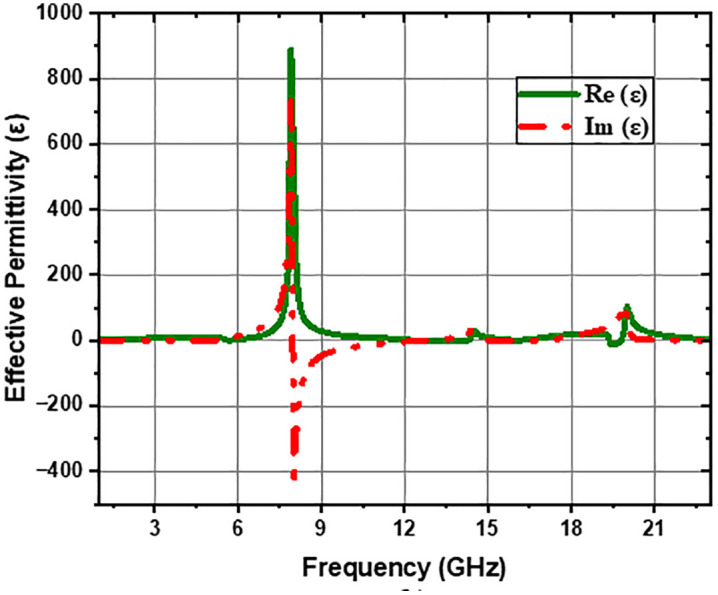
Effective permittivity of the MTM unit cell loaded with CELC.

The symbols Z, n, μ and ε represent the characteristic impedance, the effective refractive index, the effective permeability, and permittivity, respectively. The equations corresponding to these parameters were implemented in MATLAB to extract the simulation results. The simulation outcomes for the characteristic parameters of the MTM cell loaded with the CELC resonator are analyzed. Fig 4 indicates the negative real part of the effective permeability within the frequency span of 5.8 GHz to 12.4 GHz and 16.2 GHz to 20.1 GHz. The negative permeability results in the formation of an electric field associated with an evanescent wave in these frequency ranges, inducing the appearance of two wide stop bands in the intervals mentioned. These observations align with the electromagnetic and electrical simulation findings presented in Fig 3. In contrast, the Z-shaped CELC resonator [18] exhibits a negative real permeability confined to a single frequency band (6.9–8 GHz), resulting in the formation of only one stopband within the same frequency range, while the CSRR resonator, being sensitive to permittivity, provides high sensitivity for dielectric material detection [24–26]. This designed CELC-loaded MTM cell is also characterized by a mu-near-zero (MNZ) behavior in the frequency bands ranging from 1 GHz to 4 GHz and from 20.8 GHz to 23 GHz. MNZ media are generally employed as MNZ scatterers, particularly in photonic applications [27]. Furthermore, it has been demonstrated that MTMs can be utilized for manipulating hyperbolic polaritons [28]. It has also been observed from Fig 5 that the real part of the effective permittivity remains predominantly positive. However, it approaches zero in certain frequency ranges such as 1 GHz to 2 GHz, 12.2 GHz to 13.1 GHz, and 21.5 GHz to 23.3 GHz. This feature, referred to as “epsilon-near-zero” (ENZ), confers a virtually infinite effective wavelength and a group velocity approaching zero for the waves traveling through it [29]. The metamaterial has demonstrated significant potential for optimizing the performance of antennas [30–32]. It is also being exploited to reduce the number of resonators in bandpass filters, thus contributing to the miniaturization of devices [33].

### 2.3. Effective parametric analysis

**Fig 6:**
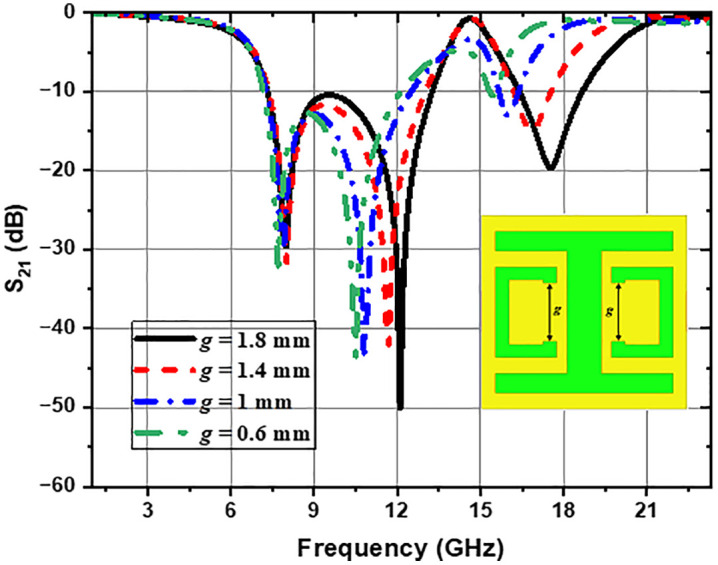
Effect of geometric parameter *g* on the CELC-loaded MTM cell.

**Fig 7:**
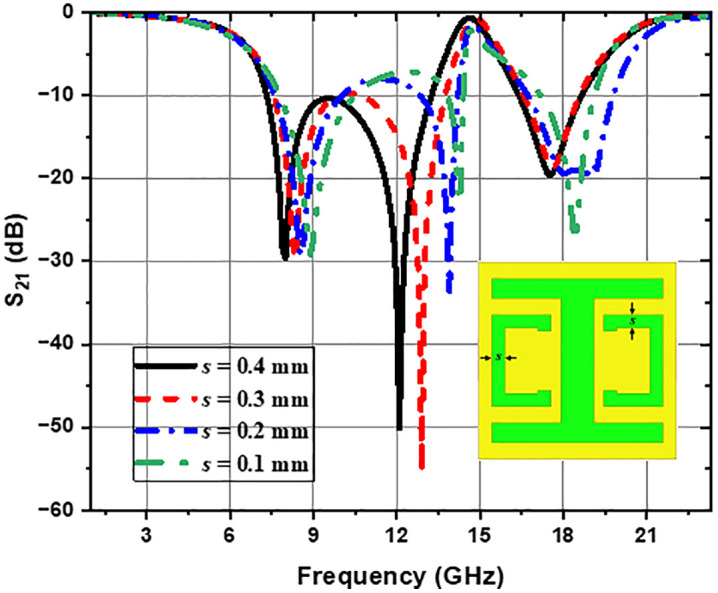
Effect of geometric parameter *s* on the CELC-loaded MTM cell.

**Fig 8:**
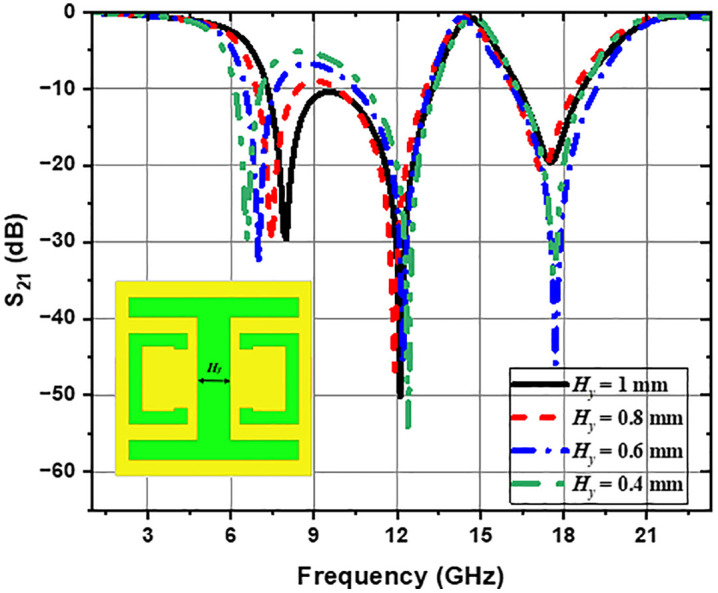
Effect of geometric parameter *H*_*y*_ on the CELC-loaded MTM cell.

**Fig 9:**
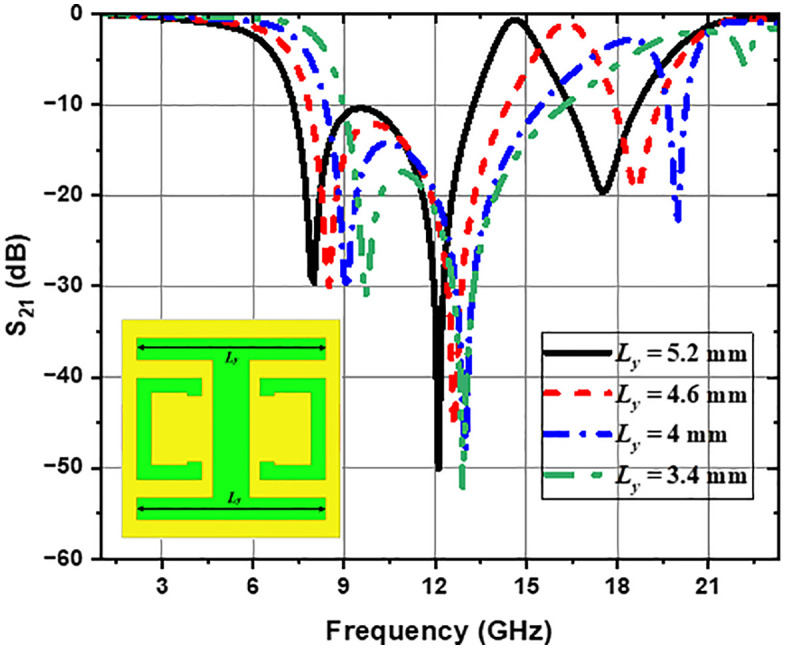
Effect of geometric parameter *L*_*y*_ on the CELC-loaded MTM cell.

The parametric study used the HFSS tool to examine the impact of geometric variations in the CELC structure on the frequency response, concerning LC resonance frequencies. Fig 6 illustrates the effect of gap variation *g* (from 1.8 mm to 0.6 mm) on the transmission parameter S_21_. It has been observed that a progressive decrease in gap length *g* induces a shift in the transmission curve towards the lower frequencies, particularly noticeable for the second and third transmission zeros (resonance frequencies), which also shift towards lower frequencies. This shift is attributed to an increase in capacitance *C*_*C*_, linked to the reduction of the gap width *g*. Furthermore, it is found that the depth of the third resonance peak intensifies as the gap *g* increases. Fig 7 illustrates the effect of varying the gap *s* on the transmission parameter S_21_. As the value of geometric slot *S* decreases, the transmission parameter S_21_ curves shift toward higher frequencies, which also causes the transmission zeros to move to higher frequency values. This shift is attributed to the increase in capacitance *C*_*C*_ and a significant decrease in the resonance frequency, so that frequency stability cannot be compensated for, resulting in a shift towards higher resonance frequencies. Furthermore, it can be noted that when *s* reaches a value of 0.2 mm, the third transmission zero is disturbed. Fig 8 shows that when the distance *H*_*y*_ decreases, the first transmission zero shifts to lower frequencies. The phenomenon can be ascribed to the increase in capacitance *C*_*S*_ due to the reduction of the slot *H*_*y*_, resulting in a decrease in the first resonance frequency to lower values. Fig 9 illustrates the impact of modifying the *L*_*y*_ length of the horizontal arms on the transmission parameter (S_21_). As *L*_*y*_ decreases, the S_21_ curves shift towards higher frequencies, also resulting in a shift of the transmission zeros towards higher frequencies. However, when *L*_*y*_ reaches a low value, the third transmission zero begins to attenuate progressively until it disappears; this attenuation increases with *L*_*y*_ reduction, ultimately resulting in only two transmission zeros for certain low *L*_*y*_ values. This is explained by the fact that a high value of *L*_*y*_ increases inductance *L*_*r*_ and capacitance *C*_*r*_, creating a longer inductive path for the current. In addition, the *C*_*r*_ capacitance between the edges of the horizontal arms increases, as a longer slot provides a larger surface area for charge accumulation and the electric field. This increase in *L*_*r*_ and *C*_*r*_ leads to resonances at lower frequencies. Conversely, the decrease in *L*_*y*_ reduces the values of inductance *L*_*r*_ and capacitance *C*_*r*_, shifting resonances to higher frequencies. The progressive disappearance of the third resonance occurs due to the excessive reduction of *L*_*r*_ and *C*_*r*_, causing this resonance to become less pronounced and negligible. For clarity, the quantitative effects of these geometrical parameters are presented in Table 1.

**Table 1:** Quantitative impact of geometrical parameter variations.

Parameter	Value	1^st^ Resonance (GHz)	2^nd^ Resonance (GHz)	3^rd^ Resonance (GHz)	1^st^ Resonance Amplitude (dB)	2^nd^ Resonance Amplitude (dB)	3^rd^ Resonance Amplitude (dB)	Observations
g	1.8 (Proposed)	8.00	12.00	17.50	30	50	20	1^st^ resonance remains unchanged; 2^nd^ and 3^rd^ resonances shift down as g decreases; the amplitudes of 2^nd^ and 3^rd^ resonances are high at g= 1.8mm
	1.4	8.00	11.72	16.75	30	32	15	
	1.0	8.00	10.98	15.75	30	33	12.7	
	0.6	8.00	10.50	15.37	30	33	11	
s	0.4 (Proposed)	8.00	12.00	17.50	30	50	20	1^st^ resonance rises slightly; 2^nd^ and 3^rd^ resonances shift up; 2^nd^ resonance amplitude decreases; 1^st^ and 3^rd^ resonance amplitudes remain nearly stable
	0.3	8.33	13.18	17.70	29	55	19	
	0.2	8.67	13.76	18.00	29	34	19	
	0.1	8.98	14.01	18.75	29	22	26	
$H_{y}$	1.0 (Proposed)	8.00	12.00	17.50	30	50	20	1^st^ resonance decreases; 2^nd^ resonance remains ~constant; 3^rd^ resonance varies slightly; the amplitudes of all resonances remain mostly stable
	0.8	7.50	12.00	17.07	29	47	21	
	0.6	6.75	12.00	17.50	29	45	47	
	0.4	6.37	12.31	17.74	33	54	34	
$L_{y}$	5.2 (Proposed)	8.00	12.00	17.50	30	50	20	1^st^ resonance rises; 2^nd^ resonance increases slightly; 3^rd^ resonance rises strongly; the amplitudes of 1^st^ and 2^nd^ resonances remain mostly stable; 3^rd^ resonance amplitude drops at $L_{y}$*=* 3.4 mm
	4.6	8.75	12.66	18.89	30	45	18	
	4	9.00	13.00	20.01	30	52	22	
	3.4	10.01	13.00	22.00	31	48	6	

## 3. Design of an ultra-wideband bandstop filter (UWB-BSF) based on CELC-loaded MTM cells

**Fig 10:**
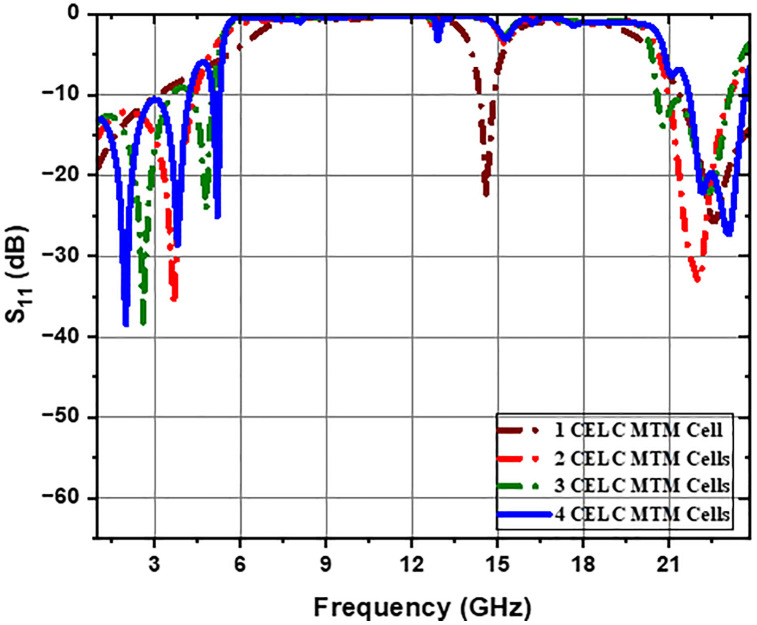
*S*_*11*_ parameter response of the cascaded CELC-loaded MTM cells.

**Fig 11:**
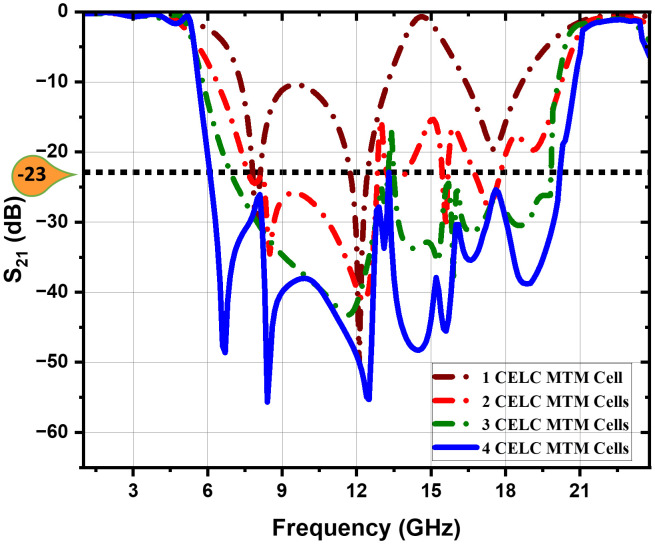
*S*_*21*_ parameter response of the cascaded CELC-loaded MTM cells.

The CELC-loaded metamaterial cell is used to design the prescribed ultra-wideband bandstop filter (UWB-BSF). To improve suppression of the transmitted signal within the specified frequency, an ingenious strategy of cascading the CELC-loaded MTM cells has been adopted. The substrate and dimensions are identical to those displayed in Fig 1, while Figs 10 and 11 show that the integration of four CELC-loaded MTM unit elements results in a UWB-BSF filter with enhanced performance. This filter offers a net attenuation of 3 dB and a band rejection of up to −55.67 dB.

The UWB-BSF response with four CELC-loaded MTM elements demonstrates significantly superior performance compared to the design with two or three elements, particularly in terms of transmission zeros and rejection levels, as observed in the *S*_*11*_ and *S*_*21*_ parameters. The S_11_ and S_21_ responses of the cascaded CELC-loaded MTM cells are shown in Figs 10 and 11, respectively. The combination of four CELC-loaded MTM elements creates a wider, deeper, and more efficient stop band. Each CELC-loaded MTM cell actively contributes to the overall suppression of the target frequency, and their combination produces a cumulative effect, with multiple resonances in the stopband as well as in the lower and upper passbands, enhancing the performance of the UWB-BSF filter. Narrow transition bands are also present. In fact, the final configuration of the proposed UWB-SBF is implemented with four CELC-loaded MTM cells. This choice is motivated by the fact that the maximum attenuation level in the stopband (S_21 _= − 23 dB) is achieved, with the generation of seven transmission zeros at 6.7 GHz, 8.4 GHz, 12.5 GHz, 14.4 GHz, 15.6 GHz, 16.6 GHz, and 18.9 GHz, along with five transmission poles located at 2 GHz, 3.8 GHz, 5.2 GHz, 22.2 GHz, and 23.25 GHz. In contrast, the use of three CELC-loaded MTM cells resulted in a maximum S_21_ of only –17.5 dB within the stopband, with five transmission zeros (11.78 GHz, 14.00 GHz, 15.35 GHz, 16.62 GHz, and 18.50 GHz) and four poles (2.80 GHz, 4.78 GHz, 20.89 GHz, and 22.50 GHz). Furthermore, the configuration with two CELC-loaded MTM cells yields a maximum S_21_ of merely –15 dB, six transmission zeros (8.45 GHz, 12 GHz, 13.54 GHz, 15.92 GHz, 17.30 GHz, and 19.30 GHz), and only two transmission poles (4.10 GHz and 22.30 GHz). It should also be noted that the use of five CELC-loaded MTM cells would significantly increase the physical dimensions of the filter, leading to a width of 30 mm, which results in considerable bulkiness and higher fabrication cost. Taking all these factors into account, the configuration with four CELC-loaded MTM cells can be considered as the most effective solution, offering superior performance in terms of selectivity, rejection level, attenuation efficiency, and compactness.

**Fig 12:**
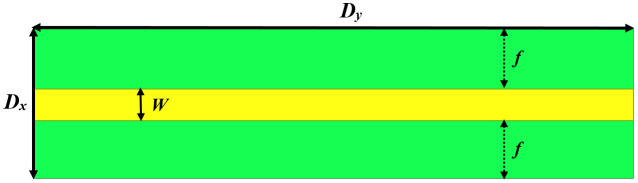
The top-view configuration of the proposed UWB-BSF.

**Fig 13:**
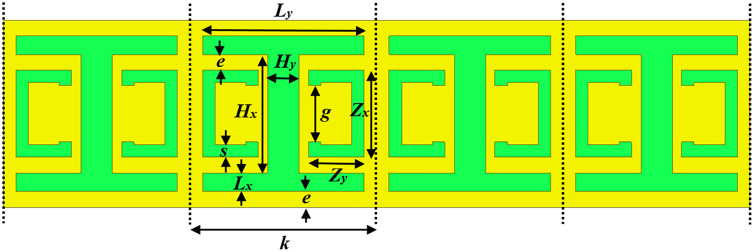
The bottom-view configuration of the proposed UWB-BSF.

**Fig 14:**
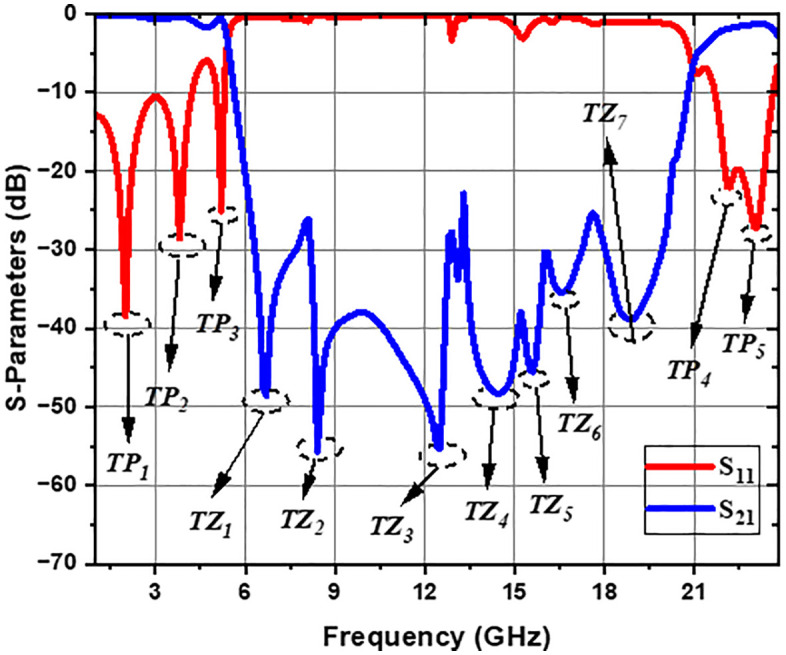
Simulated results of S-parameters for the UWB-BSF.

The top plane and bottom plane views of the proposed final ultra-wideband bandstop filter (UWB-BSF) are illustrated in Figs 12 and 13, respectively. The dimensions of the geometric parameters of the MTM unit cell and the proposed UWB-BSF model are provided in Table 2. The frequency response is shown in Fig 14. This filter, composed of four CELC-loaded MTM cells arranged in cascade and connected homogeneously, is characterized by its compact dimensions of 6 × 24 × 0.762 mm^3^ (i.e., 0.55λg×2.22λg×0.07λg) highlighting a highly miniaturized design. The exceptional selectivity of the filter is confirmed by a shape factor (SF) of 0.87, whose expression is provided in reference [34]. Such a value, close to 1, indicates a particularly rapid transition between passbands and stopbands, a crucial feature for effectively isolating unwanted frequencies. The insertion loss at 3 dB extending from 5.4 GHz to 21 GHz corresponds to a relative stopband bandwidth (RSB) of 118%, demonstrating the filter’s capability to reject an extensive range of frequencies. Insertion losses are minimal, measuring 0.67 dB at the lower passband center frequency and 1.33 dB at the center frequency of the upper passband. Furthermore, the frequency response shows seven transmission zeros distributed at 6.7 GHz (*TZ*_*1*_), 8.4 GHz (*TZ*_*2*_), 12.5 GHz (*TZ*_*3*_), 14.4 GHz (*TZ*_*4*_), 15.6 GHz (*TZ*_*5*_), 16.6 GHz (*TZ*_*6*_), and 18.9 GHz (*TZ*_*7*_), offering significant suppression of unwanted signals. Three transmission poles are observed at frequencies of 2 GHz (*TP*_*1*_), 3.8 GHz (*TP*_*2*_), and 5.2 GHz (*TP*_*3*_) in the lower bandpass, and two other transmission poles at frequencies of 22.2 GHz (*TP*_*4*_) and 23.24 GHz (*TP*_*5*_) appeared in the upper bandpass. The suppression level of *S*_*21*_ reaches a minimum of −55.67 dB and a maximum of −23 dB over the entire rejected band, confirming the filter’s high performance. These features make this UWB stopband filter an ideal solution for demanding applications, particularly in UWB domains, where suppression of harmonic frequencies and unwanted interference is essential.

**Table 2:** Geometric dimensions of the designed UWB-BSF structure.

Geometric parameters	Values (mm)	Geometric parameters	Values (mm)
$D_{x}$	6	$H_{y}$	1
$D_{y}$	24	$Z_{x}$	2.8
*W*	1.27	$Z_{y}$	1.8
*f*	2.365	s	0.4
$L_{x}$	0.6	g	1.8
$L_{y}$	5.2	e	0.5
$H_{x}$	3.8	*k*	6
Δx	6	Δy	6

## 4. Surface current and E-Field distributions

**Fig 15:**
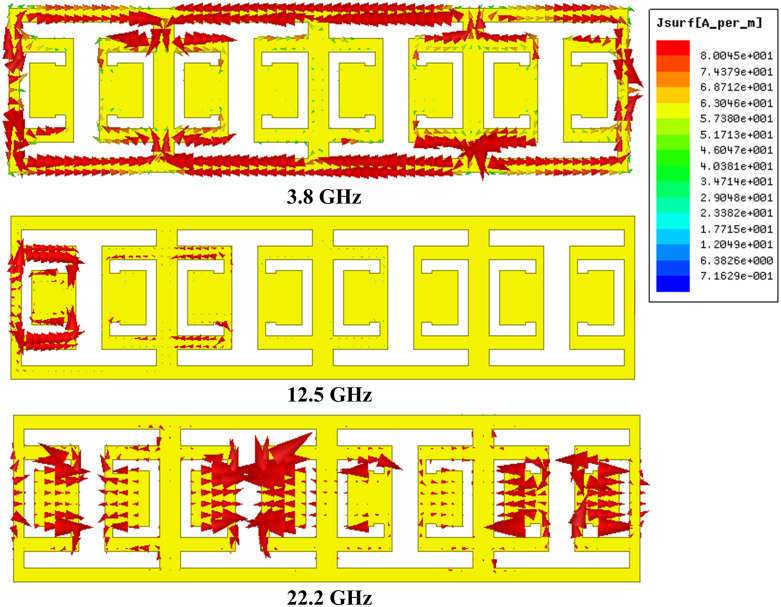
Distribution of surface current at: 3.8 GHz (lower passband frequency), 12.5 GHz (stopband frequency), 22.2 GHz (upper passband frequency).

Fig 15 depicts the surface current distribution at the resonance frequency of 3.8 GHz (*TP*_*2*_) in the lower passband. The distribution is predominantly focused around the top and bottom margins of the CELC resonators configured in an “I” form, as well as in particular areas adjacent to the CELC resonators with split-ring structures. At the frequency of 22.2 GHz (*TP*_*4*_), within the upper passband, the surface current predominantly accumulates along the peripheries of the split-ring configurations of the CELC resonators. Furthermore, at the resonance frequencies of *TP*_*2*_ and *TP*_*4*_, a particularly notable surface current intensity is detected in the vicinity of the input and output ports. This is interpreted as the transmission of the signal between the input and output ports, indicating efficient energy transfer at these frequencies. The surface current pattern at the rejected band frequency of 12.5 GHz (*TZ*_*3*_). At this frequency, the surface current is mainly concentrated inside the CELC element, with a low concentration in the half-second CELC element, located near the input port, while no concentration is observed on the third and fourth CELC elements, not near the output port. This distribution shows that the transmission energy is blocked outside the passbands, indicating that the propagation signal is not reaching the output port.

**Fig 16:**
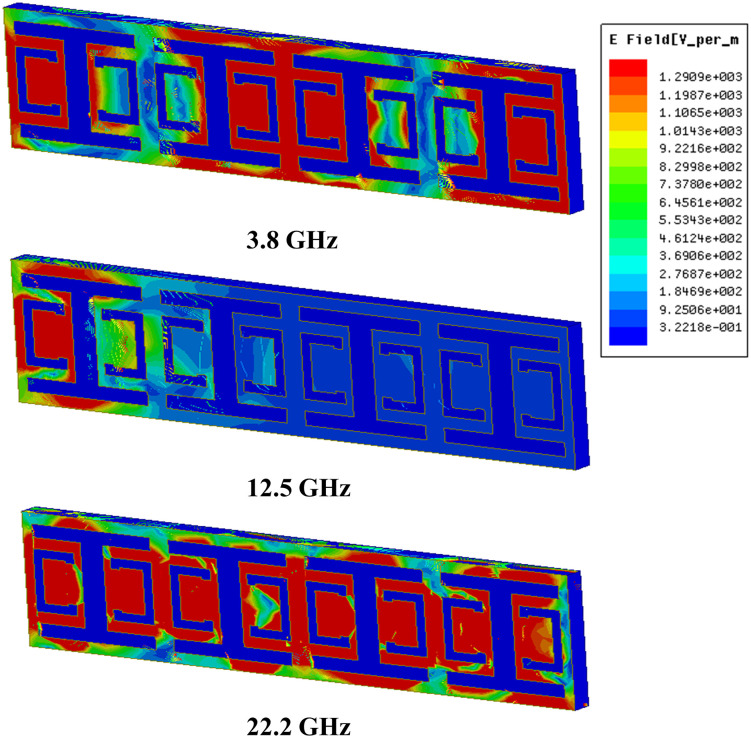
Distribution of E-field at: 3.8 GHz (lower passband frequency), 12.5 GHz (stopband frequency), 22.2 GHz (upper passband frequency).


The magnitude of the electric field (E-field) depicted in Fig 16 demonstrates that when the surface current remains constant, the electric field remains close to zero. Conversely, variations in the surface current lead to the emergence of the electric field. This behavior is attributed to the coupling between the electric and magnetic fields. These phenomena are governed by Maxwell’s equations (6,7) [35]. Equation (6) corresponds to Ampère’s law in a time-varying regime, incorporating the displacement current ($\;\frac{\partial D}{\partial t}\;$) as an additional source of magnetic field generation. Equation (7), on the other hand, describes electromagnetic induction following Faraday-Lenz’s law.

$$\nabla \times H=J+\frac{\partial D}{\partial t}$$ (6)

$$\nabla \times E=-\frac{\partial B}{\partial t}$$ (7)

In this context, $\nabla =[\frac{\partial }{\partial x},\frac{\partial }{\partial y},\frac{\partial }{\partial z}]$ denotes the Nabla operator defined in Cartesian coordinates. The vectors *D* and *B* represent the electric displacement field and the magnetic flux density, respectively. These quantities are associated with the electric field *(E)* and the magnetic field intensity *(H)* via the subsequent constitutive material equations (8, 9) [36]:


D(t)=εE(t) (8)

B(t)=μH(t) (9)

Here, *ε* and *μ* represent the permittivity and permeability, respectively.

## 5. Group delay

The degree of filtering corresponds to the group delay, which is a measure of phase distortion, as described in [37]. In microwave filters, a consistent group delay is typically found in the passband. However, Chebyshev filters exhibit a constant ripple in this band, resulting in a change in propagation time. The standard expression for calculating the group delay (*GD*) is given by equation (10).


$$GD=-\frac{\Delta \varphi }{\Delta \omega }$$ (10)

Here, ω denotes the angular frequency (radians/sec), while ϕ represents the phase angle of S_21_.

**Fig 17:**
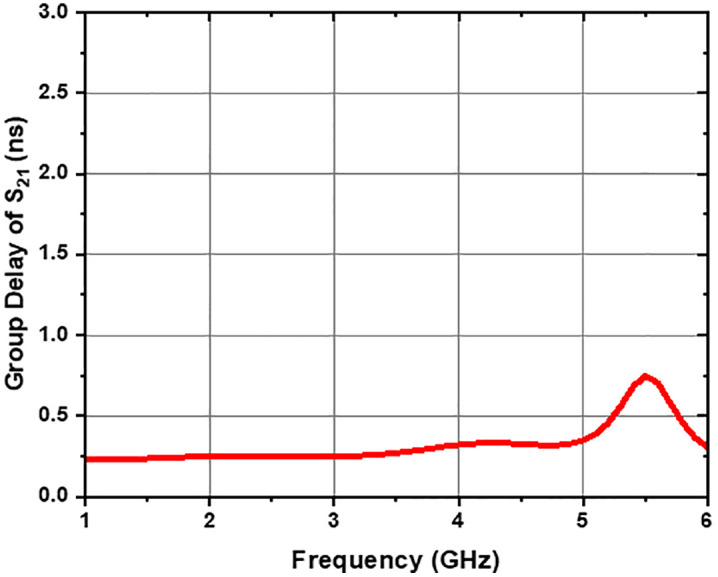
Group delay vs. frequency of UWB-BSF at lower passband.

**Fig 18:**
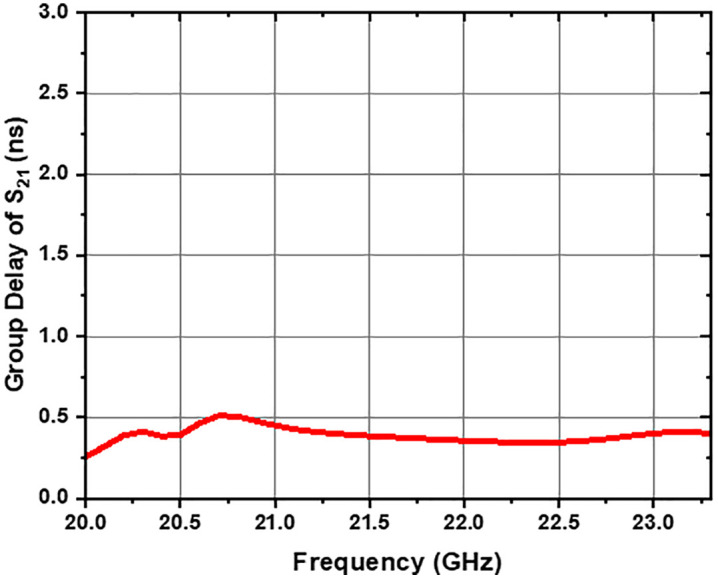
Group delay vs. frequency of UWB-BSF at upper passband.

The group delay (GD) of the UWB-BSF remains flat within the lower passband, with a value below 0.74 ns (GD ≤ 0.74 ns), as illustrated in Fig 17. Similarly, within the upper passband, the group delay also remains flat, with a value below 0.50 ns (GD ≤ 0.50 ns), as shown in Fig 18. These results indicate constant group delay across all frequencies of the signals transmitted by the filter, thus preserving the integrity of the original signal shape at its output. Indeed, relevant experimental studies on group delay measurement in similar microwave systems have already been reported in the literature [26,38,39]. It is worth noting that this measurement can be carried out relatively easily in practice.

## 6. Measurement results and discussion

**Fig 19:**
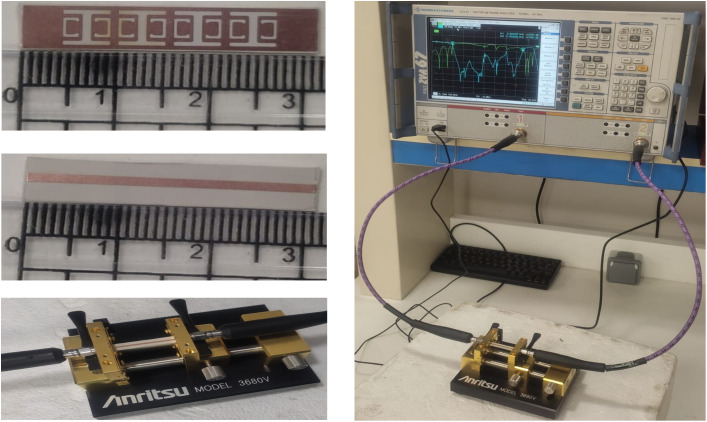
The fabricated prototype and measurement setup of the ultra-wideband bandstop filter (UWB-BSF).

**Fig 20:**
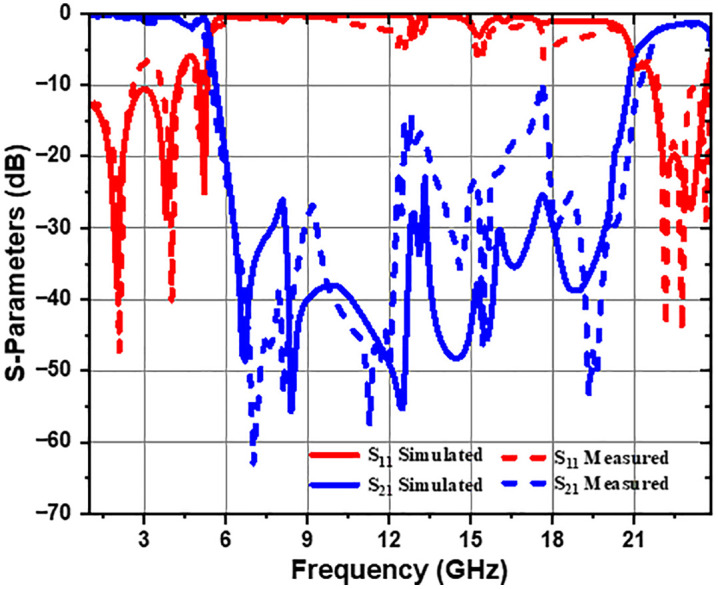
Measured and simulated results of the suggested ultra-wideband bandstop filter (UWB-BSF).

The measurement results are presented in this section. Fig 19 illustrates the fabricated filter along with its measurement setup. The measured and simulated performance of the S-parameters for the ultra-wideband bandstop filter (UWB-BSF) is reported in Fig 20. These results are obtained using the ZVA 67 vector network analyzer. The measurement instrument ZVA 67 VNA was calibrated using the TOSM (Through, Open, Short, Match) calibration method before carrying out the measurements of the proposed UWB-BSF prototype. A high consistency between the measurement and simulation results is observed. The measurements show an insertion loss 3-dB bandwidth spanning from 5.4 GHz to 21.6 GHz, indicating a relative stopband bandwidth (RSB) of 120%, with the center frequency of 13.5 GHz, demonstrating the ability of the proposed filter to provide an ultra-wide stopband. The proposed UWB-BSF is distinguished by its high compactness and extremely miniaturized dimensions, measuring 0.55λg×2.22λg×0.07λg. Seven transmission zeros are identified at the following frequencies: 6.97 GHz (*TZ*_*1*_), 8.07 GHz (*TZ*_*2*_), 11.24 GHz (*TZ*_*3*_), 14.57 GHz (*TZ*_*4*_), 15.41 GHz (*TZ*_*5*_), 18.01 GHz (*TZ*_*6*_), and 19.30 GHz (*TZ*_*7*_). These transmission zeros enhance the rejection level in the stopband and significantly improve the selectivity of the passbands. Their generation is attributed to the coupling of CELC metamaterial cells and the intrinsic characteristics of the resonators, with a shape factor of 0.87 confirming the high selectivity of the filter. The suppression level of S_21_ reaches a minimum of −62.96 dB and a maximum of −10 dB, indicating a high rejection level. Three transmission poles are observed in the lower passband, while three additional poles are identified in the upper passband. To quantitatively assess the concordance between the simulated and experimentally measured results, the relative error of some relevant parameters has been calculated and is presented in Table 3. Minor variations between the measured and simulated results are observed and quantified, mostly attributable to the parasitic effects of the SMA connectors, their related losses, and fabrication tolerances during implementation.

**Table 3:** Summary of simulated and measured results with associated relative errors.

Proposed structure	Results and analysis	RSB (%)	Rejection level (dB)	Transmission zeros (TZs)
UWB-BSF	**Simulated**	118%	−55.67 dB	6.7 GHz (*TZ*_*1*_), 8.4 GHz (*TZ*_*2*_), 12.5 GHz (*TZ*_*3*_), 14.4 GHz (*TZ*_*4*_), 15.6 GHz (*TZ*_*5*_), 16.6 GHz (*TZ*_*6*_), 18.9 GHz (*TZ*_*7*_)
	**Measured**	120%	−62.96 dB	6.97 GHz (*TZ*_*1*_), 8.07 GHz (*TZ*_*2*_), 11.24 GHz (*TZ*_*3*_), 14.57 GHz (*TZ*_*4*_), 15.41 GHz (*TZ*_*5*_), 18.01 GHz (*TZ*_*6*_), 19.30 GHz (*TZ*_*7*_).
	**Relative error (%)**	1.69%	13.09%	4.02% (*TZ*_*1*_), 3.92% (*TZ*_*2*_), 10.08% (*TZ*_*3*_), 1.18% (*TZ*_*4*_), 1.21% (*TZ*_*5*_), 8.49% (*TZ*_*6*_), 2.11% (*TZ*_*7*_)

## 7. Performance analysis with other reported BSF designs

The bandstop filter (BSF) designs reported in the literature [12,13,17,18] encompass a variety of intriguing configurations. However, a bimodal BSF based on a microstrip structure was developed using the slot line method [40]. This filter exhibits a minimum insertion loss of less than −25 dB and is notable for its compactness, which facilitates integration into electronic circuits. However, its primary limitation lies in the presence of only two transmission zeros. Another design leverages the band rejection characteristics of a split-ring resonator (SRR) [41]. This type of SRR is particularly effective for eliminating unwanted bands due to its compact size. In this approach [42], a microstrip transmission line is coupled to a Substrate Integrated Waveguide (SIW) resonator, incorporating both rectangular and curved configurations. This setup enabled the development of an efficient bandstop filter operating in the X-band, achieving significant suppression around 9 GHz with an attenuation of up to −61.9 dB. However, the selectivity of this design is limited, as it includes only a single transmission zero. A wideband bandstop filter (BSF) reported in [43] features an extended stopband along with a compact design, rendering it suitable for applications in the C and X bands. Its insertion loss in the upper passband ranges between 0.61028 dB and 1.7826 dB while offering four transmission zeros. Another design, described in [44], incorporates a compact rectangular open-loop resonator paired with a stub-loaded resonator as the core elements. This filter is characterized by two transmission zeros and a relative stopband bandwidth (RSB) of 88%.

The experimental findings validate that the efficacy of the suggested UWB-BSF surpasses the other BSFs listed in Table 4, which compares their attributes, dimensions, performance metrics, and fabrication technologies. Indeed, the filter presented in this work is distinguished by its high selectivity and its ability to effectively reject signals within a significantly widened stopband, while ensuring smooth transmission of signals in the lower and upper passbands. These features make it particularly suitable for a variety of applications within specified frequency ranges. In fact, this miniaturized ultra-wideband bandstop filter, based on a metamaterial structure loaded with a CELC resonator, is presented. An ultra-wide stopband ranging from 5.4 GHz to 21.6 GHz (RSB = 120%) is achieved, with a rejection level reaching – 62.96 dB and enhanced selectivity provided by seven transmission zeros, resulting in a shape factor of 0.87. The lower and upper passbands exhibit insertion losses of 0.67 dB and 1.33 dB, respectively. Thanks to its compact size and simplified fabrication process, the device offers a high-performance solution for interference rejection in 5G (n77, n78, n79 bands), Wi-Fi 5/6, 4G LTE applications, as well as for Ku-band satellite communications and K-band radar systems.

**Table 4:** Performance metrics compared with other BSFs.

Ref	Technology	Rejection level Min S_21_ (dB)	RSB (%)	No. of TZs	2D electrical size (λg×λg)	NCS $(\lambda_{g}^{2})$	IL (dB) in LPB/ UPB	GD (ns) in LPB/ UPB	Physical dimensions (${mm}^{3}$)	Fabrication Complexity
[6]	Planar microstrip	−68 −74	82 78	3 5	0.21 × 0.19 0.3 × 0.25	0.040 0.075	1 / 1 1 / 1	NA/ NA NA/ NA	725.19 1364.70	High Medium
[12]	Varactor diode	−60	119.4	5	0.23 × 0.32	0.073	NA/ NA	NA/ NA	347.17	High
[13]	In-line coupled lines	−60	111.8	7	1.01 × 0.93	0.93	NA/ 1.7	NA/ NA	41.60	Medium
[17]	CPW-CSRRs	−40	5.5	1	0.29 × 0.10	0.029	NA / NA	NA/ NA	NA	High
[18]	Z-Shaped CELCs	−25	28.75	1	0.33 × 1.32	0.43	NA / NA	NA/ NA	115.20	Medium
[40]	Slot Spurline	−25	10.82	2	0.35 × 1.54	0.53	NA / NA	NA/ NA	113.40	High
[41]	Slot-Type SRRs	−25	1.94	1	2.75 × 3.4	9.35	NA / NA	NA/ NA	762.00	Medium
[42]	Coupled SIW Resonator	−61.9	1.1	1	0.9 × 1.39	1.25	NA / NA	NA/ NA	NA	High
[43]	L-shaped and Quad-mode Resonator	−60	126	4	0.27 × 0.54	0.14	NA/ 1.78	NA/ NA	56.61	Medium
[44]	Open-loop Resonator	−74	88	2	0.304 × 0.304	0.092	NA / NA	NA/ NA	325.20	Medium
[45]	Lumped-element (LC-R)	−15	NA	2	NA	NA	6.72/ NA	−0.498/ NA	NA	High
[46]	SIW	−69	NA	12	NA	NA	0.8/ NA	NA/ NA	NA	High
**This work**	CELC-loaded MTM	−62.96	120	7	0.55 × 2.22	1.22	0.67/ 1.33	0.72/ 0.50	109.72	Medium

Ref*: reference; RSB*: 3-dB relative stopband bandwidth; No. of TZs: number of transmission zeros; NCS*: normalized circuit size; IL (dB) in LPB/ UPB*: insertion loss (dB) in lower passband/ upper passband; GD (ns) in LPB/ UPB*: group delay (in nanoseconds) in the lower passband/ upper passband; NA*: not available*.*

## 8. Conclusions

An ultra-wideband bandstop filter (UWB-BSF) has been designed using innovative complementary electrical LC (CELC)-loaded metamaterial resonators. The suppression of unwanted modes in the structure is accomplished via the evanescent propagation mode. The suggested filter features an ultra-wide stop band ranging from 5.4 GHz to 21.6 GHz, characterized by seven transmission zeros. Minimal insertion loss is noted in the passbands, and a shape factor close to 1 is recorded, highlighting superior selectivity. The overall tiny size (0.55λg × 2.22λg × 0.070λg) and measured performance metrics of the filter demonstrates its adaptability for modern wireless systems, including Sub-6 GHz 5G NR (New Radio), especially within the n77 (3.3–4.2 GHz), n78 (3.3–3.8 GHz), and n79 (4.4–5.0 GHz) frequency bands, as well as Wi-Fi 5 (802.11ac), Wi-Fi 6 (802.11ax), 4G LTE, Ku-band satellite communications, and K-band radar systems. Furthermore, the suggested UWB-BSF effectively addresses efficient utilization of frequency spectrums in advanced wireless systems by facilitating selective interference reduction and improving dynamic spectrum access for emerging technologies. Moreover, this UWB-BSF exhibits strong reconfigurability potential, thereby paving the way for broader applications in cognitive and agile communication systems. Two promising approaches can be considered: the integration of varactor diodes at the identified high electric-field nodes to enable dynamic adjustment of the rejection frequency, and the utilization of graphene as a tunable capacitive element, offering improved linearity and more seamless integration compared to conventional varactors. Future work will therefore focus on the design, fabrication, and characterization of these reconfigurable versions, while addressing the challenges related to accurate modelling and the linearity of active devices.
